# Microevolution of Outbreak-Associated Middle East Respiratory Syndrome Coronavirus, South Korea, 2015

**DOI:** 10.3201/eid2202.151700

**Published:** 2016-02

**Authors:** Moon-Woo Seong, So Yeon Kim, Victor Max Corman, Taek Soo Kim, Sung Im Cho, Man Jin Kim, Seung Jun Lee, Jee-Soo Lee, Soo Hyun Seo, Ji Soo Ahn, Byeong Su Yu, Nare Park, Myoung-don Oh, Wan Beom Park, Ji Yeon Lee, Gayeon Kim, Joon Sung Joh, Ina Jeong, Eui Chong Kim, Christian Drosten, Sung Sup Park

**Affiliations:** Seoul National University Hospital, Seoul, South Korea (M.-W. Seong, T.S. Kim, S.I. Cho, M.J. Kim, S.J. Lee, J.-S. Lee, S.H. Seo, J.S. Ahn, B.S. Yu, N. Park, M.D. Oh, W.B. Park, E.C. Kim, S.S. Park);; National Medical Center, Seoul (S.Y. Kim, J.Y. Lee, G. Kim, J.S. Joh, I. Jeong);; University of Bonn Medical Centre, Bonn, Germany (V.M. Corman, C. Drosten);; German Centre for Infection Research (DZIF), (V.M. Corman, C. Drosten)

**Keywords:** Middle East respiratory syndrome, MERS-CoV, coronavirus, phylogenetic analysis, genome sequencing, viruses, South Korea, outbreak, microevolution, respiratory infections

## Abstract

During the 2015 Middle East respiratory syndrome coronavirus outbreak in South Korea, we sequenced full viral genomes of strains isolated from 4 patients early and late during infection. Patients represented at least 4 generations of transmission. We found no evidence of changes in the evolutionary rate and no reason to suspect adaptive changes in viral proteins.

Middle East respiratory syndrome coronavirus (MERS-CoV), first detected in Saudi Arabia in 2012, is a novel human pathogen that causes severe respiratory illness (*1*). Phylogenetic analyses and transmission studies suggest a zoonotic origin in dromedaries (*2*,*3*). Human-to-human transmission among close contacts of patients (e.g., family members and persons in healthcare settings) has been described (*4*). As of October 2015, the World Health Organization had received reports of 1,593 cases (including at least 568 deaths), most of which were reported from the Arabian Peninsula. 

In South Korea, the first imported MERS-CoV case was identified on May 20, 2015, in a 68-year-old man who had traveled to the Middle East 2 weeks earlier (*5*). Another 185 persons in South Korea were subsequently infected during a 4-week period, mainly through in-hospital transmission. The unusually large number of cases, which occurred during at least 4 sequential generations of human-to-human transmission, raised questions regarding potential adaptations of MERS-CoV to the human host. To determine the possibility of virus adaptation, we repeatedly sequenced complete genomes for MERS-CoV from 4 patients representing different generations of transmission during the South Korea outbreak. 

## The Study

**Figure 1:**
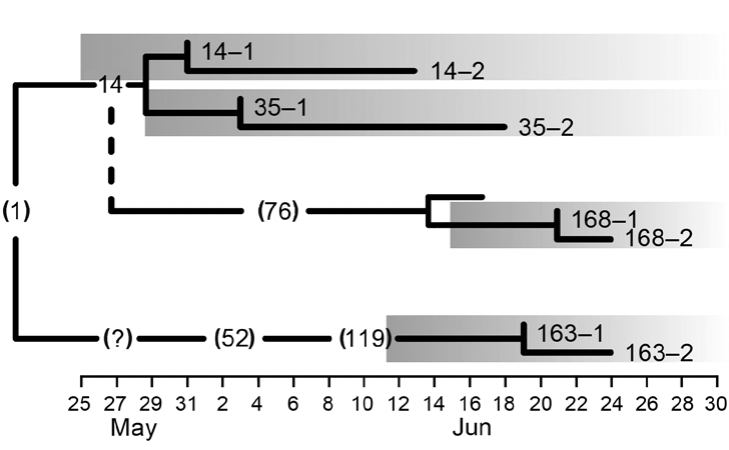
Transmission tree timeline for 8 Middle East respiratory syndrome coronavirus strains isolated during an outbreak in South Korea, 2015. Numbers without parentheses indicate patients in this study; numbers inside parentheses indicate patients not included in this study. The index case-patient is represented by (1). Numbers 1 and 2 following patient identification numbers indicate separate samples that were sequenced. The left edge of each shaded box indicates date of symptom onset for that patient; solid black vertical lines indicate sampling dates. Dashed vertical line indicates transmission from patient 14 to patient 76; (?) indicates unknown source of infection.

Institutional review boards of the Seoul National University Hospital and the National Medical Center approved this study. We tested samples from 4 patients, designated as patients 14, 35, 163, and 168 (Table 1; Technical Appendix Table 1). Patients 14, 35, and 168 were second-, third-, and fourth-generation case-patients, respectively; each had recorded exposure histories (*5*) (Figure 1). Patient 163 had a recorded transmission history that traced back to patient 119. However, for 1 interim transmission, the place and approximate time of exposure could be reconstructed, but individual contacts could not be determined. Thus, patient 163 might belong to at least the fourth, but potentially the fifth, generation of transmission (*5*).To identify virus changes, we obtained 2 samples from each of the 4 patients, 1 at the early and 1 at the late stage of infection. Clinical samples were tested for MERS-CoV RNA by reverse transcription PCR (RT-PCR) targeting the upE (upstream of E) and ORF1 (open reading frame 1) genes (*6*). Using virus quantity estimates as a basis, we determined full genomes by amplifying overlapping PCR products and sequencing as previously described (*7*). 

**Table 1:** Characteristics of cases and samples in a study of the microevolution of 8 isolates obtained from 4 patients during a MERS-CoV outbreak, South Korea, 2015*

Patient no., date of symptom onset	Sample type	Date of sample collection	Cycle threshold for upE/ORF1a	Transmission generation (source of transmission)
14, May 25				Second (patient 1 to 14)
Sample 1	ETA	May 31	24.0/25.2	
Sample 2	Sputum	Jun 13	29.0/31.5	
35, May 29				Third (patient 1 to 14 to 35)
Sample 1	Sputum	Jun 3	24.7/25.3	
Sample 2		Jun 18	27.5/28.2	
168, June 17	ETA			Fourth (patient 1 to 14 to 76 to 168)
Sample 1	Sputum	Jun 21	31.6/32.3	
Sample 2	Sputum	Jun 24	28.9/28.3	
163, June 13				Fourth or fifth (patient 1 to ? to 52 to 119 to 163)
Sample 1	Sputum	Jun 19	20.2/20.9	
Sample 2	Sputum	Jun 29	28.4/28.8	

*ETA, endotracheal aspirate; MERS-CoV, Middle East respiratory syndrome coronavirus; ORF1a, open reading frame 1a gene; upE, upstream of E gene.

Genome sequences with a minimum length of 29,831 bp (99.04% of the genome) were obtained from each specimen and submitted to GenBank (accession nos. KT374050– KT374057). All sequences clustered phylogenetically with MERS-CoV strains identified during the outbreak and with a sequence from a linked case in China (*8*,*9*). Our findings confirmed previously described evidence for recombination between MERS-CoV clades (*10*). MERS-CoV strain Hu/Riyadh KSA_2959_2015, the closest related strain outside the South Korea outbreak, was used as an outgroup to reconstruct the phylogeny of the 8 viral genomes (Technical Appendix Figure); the strain was isolated in Riyadh, Saudi Arabia, during February 2015.

**Figure 2:**
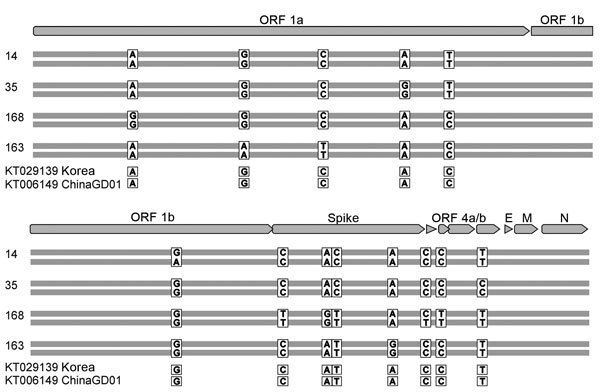
Location of the 13 variant nucleotide positions identified in genomes of 8 Middle East respiratory syndrome coronavirus strains isolated during an outbreak in South Korea, 2015. Case-patient numbers are indicated on the left, as are GenBank accession numbers for 2 related strains. For each case, the top and bottom horizontal bars represent the genome sequence generated from the first and second samples, respectively. Letters indicate matching nucleotide positions between samples. Sample collection dates are shown in Table 1. E, small envelope gene; M, matrix gene; N, nucleocapsid gene; ORF, open reading frame.

The 8 strains from South Korea shared 99.8%–99.9% nt identity with Hu/Riyadh KSA_2959_2015, deviating by 24–27 positions across the genome. Sequences for the viruses showed 13 variant nucleotide positions: 6 in the ORF1ab gene, 5 in the spike gene, and 1 each in accessory genes ORF4a and ORF5 (Figure 2). Of the 13 variants, 11 caused nonsynonymous substitutions (Technical Appendix Table 2). To analyze substitutions along the transmission tree, we reconstructed the unknown sequence of the index case-patient’s virus based on an isolate from his wife (GenBank accession no. KT029139), a virus from a patient who traveled to China (accession no. KT006149), the co-ancestral strain from Saudi Arabia (accession no. KT026453), and all sequences determined in this study. Considering that sequencing errors were possible in strains KT029139 and KT006149 due to cell culture adaptation and differences in sequencing technique (*8*,*9*), and assuming no mutation reversion within short human-to-human passage, an unequivocal ancestral sequence reconstruction was possible (*11*). The index case-patient showed overt symptoms by May 11, indicating virus exposure occurred around May 1 (*5*). This date coincides with days (May 1 and 2) that the index case-patient spent in Saudi Arabia, where, at the time, viruses most closely related to the South Korea strains were circulating (*10*). Thus, that starting sequence, as determined by ancestral sequence reconstruction, was assumed to have existed on May 1.

Substitutions observed along the transmission tree corresponded, on average, to 3.78 × 10^−6^ substitutions/site/day (Table 2). The average substitution rate within individual patients (comparing first and second samples for each patient) was not significantly different: 3.44 × 10^−6^ substitutions/site/day. The between-patient evolutionary rate was projected to be 1.3 × 10^−3^ substitutions/site/year, which is within the CI of an earlier estimate of the evolutionary rate for MERS-CoV: 1.12 × 10^−3^ substitutions/site/year (95% CI 8.76 × 10^−4^ to 1.37 × 10^−3^) (*12*). This finding suggests that the exclusively human-to-human transmission observed in this outbreak has not led to a change in the apparent evolutionary rate of the virus. No similar mutations occurred in parallel transmission chains leading up to patients 35, 168, and 163, suggesting that quasispecies sampling during transmission events has been stochastic rather than selective. Selective sampling would have been expected in the hypothetical case of emergence of a mutated virus with increased transmissibility or replication level.

**Table 2:** Substitution rates in strains within and between cases in a study of the microevolution of 8 isolates obtained during a MERS-CoV outbreak, South Korea, 2015*

Variable	Within cases				Between cases		
	Days between samples	Substitutions/ observation period	Substitutions/ site/d		Days after start of transmission chain†	Substitutions/ observation period†	Substitutions/ site/d
Patient no.							
14	13	1	2.58 × 10^–6^		30	3	3.35 × 10^–6^
35	15	0	0		33	5	5.08 × 10^–6^
168	3	1	1.12 ×10^–5^		51	6	3.94 × 10^–6^
163	10	0	0		49	4	2.74 × 10^–6^
Mean (SD)	NA	NA	3.44 × 10^–6 ^(4.59 × 10^–6^)		NA	NA	3.78 × 10^–6 ^(8.64 × 10^–7^)

*MERS-CoV, Middle East respiratory syndrome coronavirus; NA, not applicable. †Determined on the basis of the reconstructed ancestral sequence existing on May 1, 2015.

The MERS-CoV spike glycoprotein targets the cellular receptor DPP4 (dipeptidyl peptidase 4). The receptor-binding domain (RBD) consists of residues E382 to C585 (*13*,*14*). In this study, we identified 2 RBD variants: D510G and I529T. Recent mutagenesis studies indicate that alterations of key residues within this region (D510A and E513A) could substantially reduce the efficiency of binding and virus entry (*13*,*15*). Because aspartate and glycine have similar physicochemical properties, the D510G variant found in this study might resemble D510A in its potential to reduce receptor binding. 

Patient 168, a 36-year-old man who worked as a radiology technologist at a university hospital in Seoul, South Korea, carried the D510G variant virus. He was exposed to the virus via patient 76 on June 6. Symptoms developed on June 17, and he was immediately admitted to an isolation room in the hospital. RT-PCR was conducted at admission, but MERS-CoV RNA was not detected until 2 days later, suggesting an initially low level of virus replication. After MERS-CoV infection was confirmed by RT-PCR, combined interferon and ribavirin treatment was administered. The patient’s vital signs were stable, and no lung lesions were seen on chest radiographs during hospitalization. He was discharged on June 30 after 2 consecutively negative RT-PCR results (June 28 and 30). The patient’s mild clinical course and indicators of a low level of virus replication warrant further virologic study of a potentially attenuating effect of the D510G variant.

Another RBD variant, I529T, was carried by study patients 14 and 35. Although further studies are needed, physicochemical considerations suggest that this variant is unlikely to affect virus binding. Of note, patient 14 transmitted the virus to >80 tertiary case-patients and was thus considered a superspreader; however, persons infected by this patient, including patient 35, were not superspreaders.

## Conclusions

Our study is limited by the absence of full-genome information for the index case-patient’s virus and by the effect of outbreak interventions that may have concealed phenotypic virus changes. However, we found no evidence for an increase in the evolutionary rate or for adaptive changes over at least 4 generations of transmission. Changes that were observed were likely caused by transmission bottleneck effects.

Technical Appendix Clinical description of the study patients and variants and phylogenetic analysis based on variant sites identified within 8 strains of Middle East respiratory syndrome coronavirus strains isolated during a 2015 outbreak in South Korea.
