# Supplementary material for: Microevolution of Outbreak-Associated Middle East Respiratory Syndrome Coronavirus, South Korea, 2015
Source: Emerg Infect Dis. 2016 Feb;22(2):327–30. doi: 10.3201/eid2202.151700 (PMC4734539; doi:10.3201/eid2202.151700)
Supplement: Technical Appendix — Clinical description of the study patients and variants and phylogenetic analysis based on variant sites identified within 8 strains of Middle East respiratory syndrome coronavirus strains isolated during a 2015 outbreak in South Korea. [file 15-1700-Techapp-s1.pdf]

Article DOI: http://dx.doi.org/10.3201/eid2202.151700

Microevolution of Middle East Respiratory Syndrome–Coronavirus during an Outbreak, South Korea, 2015

Technical Appendix

Technical Appendix Table 1. Clinical description of the patient included in this study

Case ID	Sex	Age, y	Outcome*	Exposure history	Underlying disease
#14	M	35	Discharged	Inpatient	None
#35	M	38	Hospitalized	Healthcare worker	Fatty liver, cough-variant asthma
#168	M	36	Discharged	Healthcare worker	None
#163	F	52	Discharged	Healthcare worker	None

*Outcome status November 1, 2015

Technical Appendix Table 2. Variants identified within the eight strains

Nucleotide position Hu/Riyadh KSA_2959_2015	Gene	Nucleotide mutation compared with Hu/Riyadh KSA_2959_2015	Predicted amino acid change	#14	#35	#168	#163
2917	ORF1ab	2639A>G	Asn880Ser	A	A	G	A
5852	ORF1ab	5574G>A	Synonymous	G	G	G	A
7933	ORF1ab	7655C>T	Ser2552Phe	C	C	C	T
10089	ORF1ab	9811A>G	Ser3271Gly	A	G	A	A
11257	ORF1ab	10979C>T	Ala3660Val	T	T	C	C
18916	ORF1ab	18639G>A	Synonymous	G/A*	G	G	G
21726	Spike	271C>T	His91Tyr	C	C	T	C
22984	Spike	1529A>G	Asp510Gly	A	A	G	A
23041	Spike	1586T>C	Ile529Thr	C	C	T	T
24622	Spike	3167A>G	Gln1056Arg	A	A	A	G

Nucleotide position Hu/Riyadh KSA_2959_2015	Gene	Nucleotide mutation compared with Hu/Riyadh KSA_2959_2015	Predicted amino acid change	#14	#35	#168	#163
25495	Spike	4040C>T	Pro1347Leu	C	C	C/T*	C
25903	ORF4a	52C>T	Pro18Ser	C	C	T	C
26979	ORF5	140T>C	Val47Ala	T	C	T	T

*These nucleotide changes (G to A and C to T) appear during the course of infection.

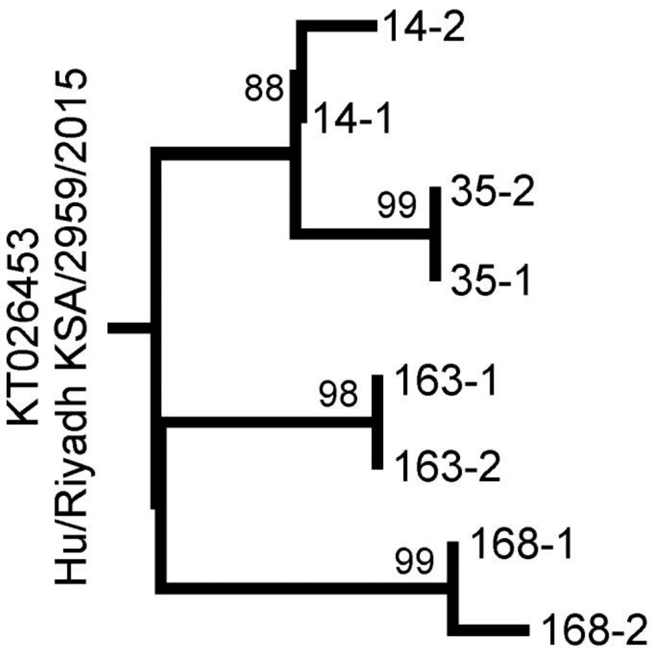

Technical Appendix Figure. Phylogenetic analysis based on variant sites using maximum likelihood (PhyML) with a general time reversible substitution model. A total of 1,000 bootstrapped phylogeny reconstructions were performed. The tree is rooted to a MERS-CoV sequence obtained from a recent outbreak in Riyadh/KSA (GenBank accession no. KT026453), which was found to constitute the closest phylogenetic neighbor to the viruses shown here (strain reference shown at the tree root).
